# Being able to think when caught in the maelstrom - how adolescents used mindfulness when facing exams

**DOI:** 10.1080/17482631.2024.2375660

**Published:** 2024-07-05

**Authors:** Ingrid Dundas, Per-Einar Binder

**Affiliations:** Department of Clinical Psychology, Faculty of Psychology, University of Bergen, Bergen, Norway

**Keywords:** Evaluation anxiety, test anxiety, mindfulness-based stress reduction, adolescents, qualitative research, high school, thematic analysis, attentional focus, unwholesome thoughts, cognitive

## Abstract

**Purpose:**

Research indicates that exam anxiety may decline with mindfulness-based interventions but there is a lack of research on adolescents’ accounts of the processes involved. We explored high-school students’ descriptions of how they perceived and applied mindfulness in managing anxiety-inducing thoughts related to academic performance following an 8-week Mindfulness-Based Stress Reduction (MBSR) course.

**Method:**

Post-course individual semi-structured interviews with 22 high school students (2 males, mean age 17.8 years) were transcribed verbatim and analysed using reflexive thematic analysis.

**Results:**

The analyses identified six themes: (1) Noticing and attending to the attention-binding “maelstrom” of anxious thoughts and feelings (2) Attending to the breath to cope with the maelstrom, (3) “removing” and “getting rid of” anxious thoughts (4) Being able to “think” (5) awareness of more helpful thoughts, and (6) Agency and control. The findings are discussed in light of the Buddhist notion of “unwholesome thoughts” and the distinction between thought suppression and the use of breathing as a benign distraction. We propose that mindfulness encompasses both a receptive, nonjudgmental awareness and an active, intentional redirection of attention.

**Conclusion:**

Mindfulness training aided participants by enhancing their capacity to disengage from fear-engaging thoughts, thereby maintaining them within their window of tolerance and facilitating cognitive processing.

Maintaining focus and cognitive clarity during anxiety-inducing situations, particularly in the context of stressful academic evaluations like exams, presents a significant challenge. Test anxiety encompasses both affective and cognitive components (Zeidner, 1998). The affective components may manifest through emotions of panic and fear, alongside physiological reactions including trembling, upset stomach, and excessive sweating. On the cognitive side, individuals may experience intrusive thoughts that disrupt their ability to access and utilize previously learned information during critical moments such as presentations and examinations. Adolescents experiencing high levels of test anxiety are more likely to report off-task thoughts, such as wishing for the situation to end, and negative self-assessments, such as doubts about their performance, compared to their less anxious counterparts (King et al., 1995).

Mindfulness has been defined in part as “the self-regulation of attention, which involves sustained attention, attention switching, and the inhibition of elaborate processing” (Bishop et al., 2004, p. 233). Practices commonly include directing attention to moment-to-moment sensations of the breath, bodily sensations, or other stimuli. This is done non-judgingly, allowing thoughts, images, and feelings to enter the mind without judging them as right or wrong. Mindfulness also involves non-reactiveness which refers to “the ability to allow thoughts and feelings to come and go, without getting caught up in or carried away by them” (Baer et al., 2008, p. 330).

This study builds upon previous research by examining interviews with high school students about their perceptions and applications of mindfulness in managing anxiety-provoking thoughts related to academic performance. Existing evidence suggests that mindfulness-based interventions (MBIs) can mitigate test anxiety among university and college students (Cho et al., 2016; Dundas et al., 2020; Lothes et al., 2023; Priebe & Kurtz-Costes, 2022). Additional studies indicate that mindfulness may be helpful for younger age groups with test anxiety (Gouda et al., 2016; McLeod & Boyes, 2021; Pires et al., 2023; Zandi et al., 2021). However, there is a lack of research on how adolescent participants perceive and apply mindfulness specifically in relation to tests and exams. While qualitative research exists on college and university students’ experiences with mindfulness interventions for evaluation anxiety (e.g., Hjeltnes et al., 2015), to our knowledge, no studies have explored the experiences of younger students. Younger students are typically less familiar with mindfulness discourses and might utilize mindfulness differently than older students. They might have different, age- and context-related challenges concerning school, peers, and exams. To further develop and adapt mindfulness-based interventions in working with test-anxious adolescents, it is beneficial to understand mindfulness and its uses from adolescents’ perspectives. This might also enhance our understanding of the processes involved. Such knowledge might inform adaptations of MBI’s to this specific group.

There are numerous theories and research findings on how mindfulness might benefit a wide range of conditions. Many of these theories are relevant for coping with test anxiety. These theories generally converge on two main proposals: that mindfulness might help students face rather than avoid the discomfort of an upcoming test, and that mindfulness might help students cope with distracting emotions and thoughts. For example, theories propose that there is a “window of emotional tolerance” for optimal functioning (Siegel, 1999). This refers to the phenomenon that there are levels where physiological and emotional arousal is too high or too low for individuals to function adequately. Mindfulness practices might help individuals regulate their emotional state to remain within their window of tolerance under stress (Siegel, 1999).

A related idea is the suggestion that mindfulness may improve the ability to allocate cognitive resources to test-relevant topics even when faced with disturbing anxiety (Dunning et al., 2022, p. 111). Mindfulness trains the ability to acknowledge disturbing anxiety-provoking thoughts but then to let them “pass as events in the mind” rather than get carried away by them (Kabat-Zinn, 2005). Finally, mindfulness training might also replace habits of thought avoidance, enhancing the ability to face troubling thoughts about exams (McCluskey et al., 2022).

We aimed to investigate how high school students describe applying mindfulness strategies to navigate thoughts that provoke anxiety concerning academic performance. We analyse and attempt to integrate students’ descriptions and theories with our theories and readings on mindfulness, thought suppression, the window of emotional tolerance, and the Buddhist notion of unwholesome thought. In doing this, we hope to identify potential underlying processes that could explain how participants can utilize a mindfulness-based intervention to deal with their evaluation anxiety.

## Method

### Participants

The study was part of a series of studies on evaluation anxiety and approved by the Regional Committees for Medical and Health Research Ethics in Norway (2009/2590), which means that it is per the Helsinki Declaration. The current study involved participants from a mixed methods study on mindfulness-based stress reduction (MBSR) for high-school students. Participants for the study were recruited via information on the web page and posters at three local high schools, and a newspaper ad. They were recruited the semester before each course since the study required a registration of state anxiety before an end-of-semester exam before the courses began. Forty-three students contacted the researchers and underwent a screening for eligibility. Exclusion criteria were needing more extensive treatment because of serious psychotic symptoms, post-traumatic symptoms, substance abuse, flashbacks, eating disorders, or suicidal plans. Participants were also screened a second time immediately after the Introduction meeting the next semester. All participants were informed that they could contact the facilitators should they experience problems during or between sessions. During the screenings, three students mentioned moderate disordered eating, psychosis-like experiences, and former suicidal plans. These are problems that are not uncommon among stressed adolescents. However, they had family support and support from the healthcare system, so they were included in the study. Three other potential participants were referred for, or found, other treatment.

Thirty-three adolescents presented for an MBSR information meeting the next semester and enrolled in one of three 8-week MBSR courses offered during the two-year study. Twenty-two participants completed the intervention. Twenty were available for interviews. Two non-completers were also available, totalling 22 interviews (2 males, age range 16–25 years, mean age 17.8 years).

### The researchers

The researchers have worked as clinical psychologists, university teachers, and qualitative and quantitative researchers for approximately 30 years each. They have long-term personal mindfulness practice and experience in conducting mindfulness courses for research purposes. The first author was trained in mindfulness at Seattle Insight Society and by Kabat-Zinn’s group (Kabat-Zinn, 2005), during a sabbatical stay in 2007 and for shorter visits to the USA later. She was one of two facilitators of the courses and did the initial inductive coding of interviews. The second author is a professor in clinical psychology with training in experiential, psychodynamic, and mindfulness-based therapy, broad experience with qualitative research methods, and interest in existential approaches within theoretical and philosophical psychology. He participated in the analyses by reading the transcribed interviews in full and repeatedly discussing the theme structure with the first author. He also participated in the interviewing and writing-up of the results.

### The intervention

The intervention consisted of weekly 2.5-hour sessions for 8 weeks, homework assignments, and a retreat, as per the standard MBSR protocol (Santorelli & Kabat-Zinn, 2009). All meetings took place at the local university. The retreat was scheduled on a Saturday and lasted four hours. The courses were held in the evenings after school. The MBSR course is structured and highly experiential and teaches practices such as sitting and walking meditation, body scan, and mindful movement (simple hatha yoga). Several practices involve attending to the sensations of the breath from moment to moment. The purpose of this practice is to anchor attention to the present moment. Participants are instructed to notice as thoughts wander off to past and present, worries and regrets, and to bring attention back to the breath in a friendly way each time this happens. Participants are also introduced to a short practice called the “breathing space”, which includes a similar breath focus and can be used whenever stress arises. After each practice, there is an “inquiry” part where participants are encouraged to share their experiences. In this study, inquiries and discussions were influenced by the participants’ occupation with school-related stressors. The psycho-educational parts of the program were adapted to use school-related stress as examples.

### Interviews

Within a month after the course, all 33 high school students who originally signed up for the courses were invited to semi-structured interviews. Twenty of the 22 completers and two non-completers were available. The interviewers were the second author, two other psychologists, and one graduate psychology student. None were involved in conducting the intervention.

The interviews started with a statement that many people were unfamiliar with the amount of stress often encountered in high school and asked participants about their own experiences (for the interview guide, see Appendix). A general question about how they experienced the intervention was followed by probes about any helpful or challenging aspects. If participants did not spontaneously mention tests and exams, interviewers asked about this. Interviewers followed Kvale’s advice for conducting interviews, for example checking one’s understanding by rephrasing and asking further, so that the interpretation in effect begins with the participant present and the audio recorder still on (Kvale, 1987, 1988, 1994a, 1994b). To further decrease the risk that interviewers and participants were talking past each other, interviewers asked for concrete examples (Haavind, 1987). Two of the three interviewers did not have intimate knowledge of the MBSR program and were transparent about this. Interviewers were asked to avoid introducing concepts specific to mindfulness discourses and to use the participants’ own words.

### Analysis

Interviews were transcribed verbatim by university students and psychologists. Transcripts were analysed from a reflexive thematic approach (Braun & Clarke, 2006, 2019, 2021a, 2021b). Braun and Clarke (2021b) underscore the importance of transparency regarding one’s paradigmatic and epistemological assumptions, noting that there are many types of thematic approaches with differing quality criteria. We place ourselves slightly more towards the critical realist end of the interpretive/constructionist—essentialist/realist dimension, seeing our influence as researchers as both unavoidable and a resource. We aim to understand the participants’ experiences from their point of view as well as our own. We believe interviewers and participants co-create meanings during the interviews and after. Since our interpretations of participants’ descriptions go beyond what participants explicitly expressed, we see the need to continually reflect upon our contributions in interpreting and developing the findings (reflexivity).

The first author went through all interviews several times using the program NVivo to code all interviews inductively, choosing labels for each meaning unit as she worked through the text. She coded and recoded the material iteratively, using the steps suggested by Braun and Clarke (2006). The resulting coding structure was discussed and finalized together with the second author.

## Results

The analysis resulted in six themes relevant to the research question of how high-school students applied learnings from the course to deal with anxiety-inducing thoughts related to academic performance. The themes all reflect ways of dealing with test anxiety that were described as new and related to the course. They are similar in that they describe issues related to awareness and attention. The names below are pseudonyms.

### Noticing and attending to the attention-binding maelstrom of anxious thoughts and feelings

When asked about their situation before the course, participants described symptoms of stress such as crying, vomiting, and trembling. Typically, “to stress” and “having a lot of thoughts” were used to describe being anxious. Their coping attempts encompassed trying not to think about exams and/or hectic and fearful cramming. The attempts to avoid thinking about exams could make matters worse:

I didn’t want to prepare because then I would start thinking about it [the exam]. As a result, things actually get worse and worse the more one comes close to [the exam] and the more one hasn’t made any preparations. (Alice)

Bert referred to anxious and hopeless thoughts as “the negative maelstrom of thoughts that just sucks all positivity into itself”. We see this as illustrating the chaos and pull they represented. After attending the intervention, participants seemed to have an increased ability to face this maelstrom of anxious thoughts and feelings without being carried away. Cecilia explained: “I think it has become easier for me to notice as soon as I start to get carried away (…) Earlier, I would not notice it, it just continued and continued”. Similarly, Dina said that she increasingly managed to “discontinue” painful streams of thought, after the course. She stated that she was more able to notice when her attention was caught in unhelpful streams of thought, and consciously discontinuing that stream. She explained:

I manage in a way to catch myself in the act of thinking those [worried] thoughts much earlier than I used to. And then to stop thinking so destructively. (…) I don’t go totally down in the cellar like I did before.

### Attending to the breath to cope with the maelstrom

Being able to redirect attention to the breath was contrasted with being swept away by troubling thoughts and feelings:

Earlier I would just have continued and continued [to be caught in the flow of anxious thoughts], and just follow along with those thoughts until. and just flowed with the thoughts. But now I’m able to. I can stop and look at it a bit from the outside and, yes, look at it from a wider perspective, in a way. (Elisabeth)

Alice noted: “When I *notice* that I’m stressing, it is now more possible for me to [check]: am I breathing?” Cecilia said she could direct attention to the breath and “stop before the feelings start to take complete control”. Fiona said that when she directed attention to her breath, “her head” was occupied with the sensation of the breath. and “filled with that, instead of being filled with all the stressful thoughts”. Greta explained:

Everything that stressed me, everything I dread, comes [flowing during exams] (…) and then I breathe a little, and then things are all right. Or they are better, at least. (Interviewer: So, you breathe and focus on the breath). Yes, and then I forget things that are around me, and when I’m done focusing on the breath, I open my eyes and am fine, so then I feel calmer.

### “Removing” and “getting rid of” anxious thoughts

Some participants used the expressions to “get rid of” and “remove” distressing thoughts. Dina said the practices would “remove it” [the disturbing thoughts], so that “you don’t’ have something always going on, in your head”. Although attending to the breath didn’t always totally remove troubled thoughts, it reduced their influence so that she would be less affected by them, according to Fiona:

[before a test] I attend to my breathing, and concentrate on that [the breath], so much that I don’t think about everything else [worries]. And that makes you more relaxed.

Hazel said that when attending to the breath, the anxious thoughts would at first keep on coming. However, soon more encouraging thoughts would find room in her mind, and she would fix her attention on those instead of the discouraging thoughts. Attending to the breath was described as turning attention to something safe and calming.

the breath, in itself, is not stressful (…) it’s something that I know is there, that always returns, and that it continues in a., it’s always there with me. And if I focus on it, there is a kind of *safety* in that.

### Being able to think

Sometimes participants described changes after the intervention as being “able to think”. Iris explained that she could easily lose her ability for rational thought when anxious: “When I’m stressed, I don’t manage to think rationally, and then I easily feel hopeless and start crying”. She said that she used to feel that troubling thoughts would crowd her head so that she was unable to work on the test. As a result, she would feel hopeless and tearful. However, the practices helped her attend to the academic material, aborting the stream of overwhelming hopelessness. She explained:

I used to feel so stressed that all thoughts would come rushing at the same time (…) and it felt impossible to solve the questions on the test. [Clears throat] Because I would think “This will never work”. And in situations when I feel that much stress, I’m [typically] unable to *think* rationally and more likely to feel that hopelessness and start crying. But now, at times when I’m stressed, it doesn’t get that far. (…) it doesn’t get to that point where everything feels hopeless, and I just wanted to dig myself down into the earth. [escape everything]

Cecilia said her thoughts used to be locked in a fear of what could happen:

I would think that this is just going totally bad, and my grades will be terrible, and everything [all my thoughts] circled around what would happen later, (…) but now, I think to myself: I’m doing what I can, and that’s good enough.

The ability to think could be facilitated by looking at the troubling thoughts and feelings as if from a distance:

It [mindfulness] is useful if something is … bothering me, bothering me so much that I’m unable to do the things I want to do. Then it’s nice that I can position myself a bit away from it, just look at it from the outside, and be calm. And then, when you are calm and can handle it, you can enter, and actually complete, what you are doing [the academic task]. (Fiona)

Jane described stepping out of streams of anxious thoughts by looking at those thoughts as “events in the mind”:

It’s been quite nice. When you look at your thoughts simply as events in the mind, - [seeing] that they are not the truth, that no one can write them in stone, so to say, - then it’s been a part of getting out of those streams. [of troubled thoughts]

The mindfulness practice of taking a breathing pause helped Dina discover that she could redirect her attention towards her assignments during exams:

It’s like I get a new way of looking at the assignments. I stop thinking solely about what can go wrong. [instead I] just think about what I need to do. (…) Interviewer: is this different from what you used to do before the course? Dina: Yes absolutely. Earlier I really used to, like I said: panic. I would feel: “I’m not managing any of this, I’m not managing any of this”. (...) so, yes, absolutely: totally different.

### Awareness of more helpful thoughts

It seemed that attending to the breath and freeing cognitive capacity (being able to “think”) made it possible for more helpful thoughts to emerge, and to be noticed. This included discovering knowledge that had been lost during high arousal. Cecilia described academic knowledge becoming available during exams:

[by doing a mindfulness practice and] just being able to relax completely, and then everything comes [to mind], then everything I have learned comes, sort of. So, it [tests] has gone very well. (…) I notice that I become very calm, I sort of don’t …, [get caught in troubling thoughts] I think to myself that “this will go well”. When you meditate, you get … it kind of gets you “here and now”, I’m not other places, [I remind myself] “now I’m writing, I’m going to concentrate here and now, *only on this subject matter*.” (…) So I don’t let my thoughts wander to other things.

Elisabeth described how oral presentations used to lock her into a state of panic. However, lately, she has discovered that she could manage to “think” during presentations rather than just deliver a prepared text. Having more cognitive capacity available, she was even able to consider that the glances she got from others during a group presentation might not be as disapproving as she used to believe. She reminded herself that her beliefs were just thoughts and not necessarily accurate:

and I just let thoughts be thoughts, so that the strange glances that I get from others watching, they don’t really matter because it’s just me imagining that they are negative [disapproving] (…) and I’ve managed to relax a bit more when I’m up there presenting, because I manage to *think* a bit rather than just deliver a rote text. (…) I’ve gotten rid of some of the stress and some of the tremor and stuff. So that when it’s my turn to speak, so eh, I’ve managed to stay much calmer. (…) [Before] I would just stand there and worry and wait until the next time I had to speak, and just stand there listening to how clever the others were and look at the kids that we were speaking to, and saw that they just were not listening - or just looking a bit strangely at us - and stuff. While now, when it’s not my turn to speak, I just look towards the floor, instead, and just focus on my breathing and myself. I notice that I manage to perform much better now (…) I don’t care as much as I did before about the judgments [of others] or negative things, and so on. But I notice much more all the things that are positive (Interviewer: can you give an example?) (…) Things that make me happy.

Doing a short mindfulness breathing practice also helped participants *work with* difficult feelings. Fiona explained that when being able to take a step back and look at her feelings:

it’s easier to *see* [discover] things (…) you have to think a bit more about what you are feeling, (…) so that you also can, sort of, you have to consider them [your feelings] a bit, you cannot just let them be there and pretend that they are not there. They bother you while they are there. And if you can take them out [into the light] and look at them a bit, then maybe they are not as [bad] as you thought. And sometimes they are much worse than you thought, then you need to think a bit more about them.

So, participants described their practices of turning attention away from troubling thoughts by attending to the breath as enabling them to notice other, more helpful, thoughts. Noticing these thoughts could also mean actively encouraging them. For example, Dina said that after the course, she would find it possible to actively notice when more helpful thoughts would enter her mind, such as “It’s alright [her name], you’ve done your best”. She actively let her attention *stay* with these more helpful thoughts, letting the worried thoughts go. She said that this prevented her from being swept away by worried thoughts.

### Agency and control

Being able to relocate attention was also described as “pulling oneself back again” (from distracting troubling thoughts) and “staying present”. Dina said that she had improved her ability to relocate her attention when it had been usurped by unhelpful thoughts during lectures. She explained: “When you are seventeen it’s not at all that easy to focus on one thing all the time (laughs). But, yes, I would say it has increased. My ability to stay present has increased, at least a bit”. Redirecting attention required making choices. Alice said that practicing the body scan had taught her how to do this. The body scan involves wilfully noticing each body part in sequence, moving attention to the next body part when instructed to do so. Dina said that she had learned that it was possible to “focus on other things, even if something else is bothering you”.

Greta said that the practices helped her “to focus on that which I am supposed to focus upon, and not on everything else around me that stresses me”. Alice said she was able to be more “present” with the topic she was studying because she had become more able to notice “when I [my attentional focus] is no longer there”. Dina explained that learning mindfulness practices provided a sense of agency and reduced the amount of stress she experienced:

I’ve developed a better ability to cope with stress, and then [as a result] the amount of stress is reduced. So, it’s two aspects of the same thing. For me, at least. I do still become stressed, but I’m more skilled at coping with it, and as a result, there is less stress.

Hazel noted that turning her attention to the breath involved turning attention away from something she could *not* control—her stress response, - to something she *could* control. She stated: “I’ve moved the focus from that which was stressing me, over to something that I can control”.

Redirecting attention from troubling thoughts was sometimes referred to as drawing oneself out of “autopilot”. This term refers to automatic, habitual, or unconscious responses. Drawing oneself out of autopilot implied having choices. Greta explained:

You can get yourself out of that autopilot that you may be on. If you are going along [with a string of worries], you can stop a bit. (…) if you’re on some kind of autopilot and not present, it’s good to just get pulled out of it, or manage to pull *yourself* out of it, I would say.

Deciding whether to attend to the breath or the stream of thoughts was described as a conscious choice: “I can choose, consciously, whether I want to concentrate on the breath, or if I shall let myself get carried away”. Kevin described that doing a short mindfulness practice during exams helped him to think more clearly and to recognize his choices:

My breath slows down and I stop thinking [of] very rash solutions. I kind of get to see the bigger picture. You get to take a step back, you’re not totally into the situation all the time, you kind of get to take a step back, look around a bit, what are your choices, what are your options, breathe a little. When you’re in the middle of it all, it’s very difficult to see all your options and to think clearly.

Lisa elaborated on how the present moment focus on her acts and thoughts enabled her to realize that she could choose what to do:

It’s about being focused on what you are doing at this moment, the thoughts you have, and the choices you make concerning those thoughts. That’s something that I remind myself of, quite often: so long your choices are with awareness, it doesn’t matter what your choice is. As long as you are doing it [making choices] with awareness rather than on autopilot.

According to Cecilia, practicing a mindful break when anxiety and worries entered the mind, was described as stopping before overwhelming feelings would “take complete control”. This was contrasted to how it had been earlier:

I’ve been stressed and, extremely stressed, and then I become just frustrated and aggressive and sad about everything, just letting the feeling swirl around. Now, it’s easier to actually stop before feelings start to take complete control. [Interviewer: How do you manage to stop?] it’s like, I try to just see what I can. what I can do, about this situation, and what’s best for me right now, [and] use the practices that I’ve learned (…) it becomes easier to not lose your focus. It actually brings attention back to what’s important to [focus on]

This ability could be useful during exams. Monica explained:

Particularly during written exams, sometimes when I notice that I’m very stressed and stuff, I’ve managed to just direct attention to the breath and held it there for a little while, and then I’ve continued, being able to continue with the exam.

## Discussion

Participants described their ability to face anxious thoughts when attending to the breath. As a result, they were able to “think”, to notice more beneficial and hopeful contents of the mind, and have a choice of stepping out of autopilot. In describing their experiences, they sometimes used expressions that are uncommon in mindfulness discourses, such as pushing away and removing anxious thoughts. In the following discussion, we aim to uncover possible underlying processes based on their descriptions. According to Gadamer’s and others’ work, a fusion of horizons can happen through dialogue, as during interviews or when interpreting a text. As in hermeneutic approaches in general, the results reflect a combination of our own and the students’ “horizons of understanding” (George & Heiden, 2022; Spence, 2017).

### “Removing” anxious thoughts, is that mindfulness?

As mindfulness instructors and researchers, we approached claims of removing and getting rid of anxious thoughts with caution. The concept of getting rid of anxious thoughts may appear antithetical to mindfulness, wherein all thoughts and feelings are permitted to enter the mind without judgement or resistance. Trying to keep certain thoughts out of awareness may seem like thought suppression, which is unhelpful in relation to academic tests (Imhof & Schulte-Jakubowski, 2015). Thought suppression requires cognitive resources to keep unwanted thoughts out of the mind (Wegner et al., 1987). The theory of Self-Referent Executive Functioning (S-REF) suggests that trying to combat anxiety-provoking thoughts about oneself (e.g., I am doing poorly) maintains test anxiety (Zeidner, 1998; Zeidner & Matthews, 2005), implying that the ability of an intervention to reduce such negative self-referential thoughts might be central in understanding how interventions can reduce test anxiety.

Participants in our study explained that they used to be haunted by anxious thoughts during preparations and the exam. This was described as a “maelstrom”, which “continued and continued”. We found this to be an interesting metaphor since a maelstrom is a rapid swirl of force that sucks everything into itself, difficult to break free from, that may carry you away and drown you. This illustrated to our mind how well anxiety may usurp both cognitive and affective resources and bind attention like a maelstrom.

The thoughts that carried one away, e.g., thoughts about being doomed and not being capable of managing the exam, are both self-referential and focused on a threat. Generally, there is a tendency for self-related thoughts to take priority in working memory (Yin et al., 2019). The same holds true for thoughts related to a threat. Such thoughts trigger attempts at self-protection: fleeing or fighting. Thus, it seems reasonable to expect that the thoughts that participants described in the current study would take up space in working memory.

How can individuals effectively manage such self-referential, intrusive thoughts? Wegner (2011) posits that focused self-distraction is more efficacious than thought suppression for dispelling unwanted thoughts. This method entails directing attention towards a specific distractor, such as visualizing a red Volkswagen, whenever intrusive thoughts arise. Wegner (2011) has suggested that the breath may serve as an effective focused distracter (p. 674).

We suggest that using the breath as a beneficial distractor describes exactly what participants spoke of. For example, one participant said that “filling the head” with [sensations of] the breath could replace stressful thoughts. This seemed to imply that the head was a container that could hold just a limited number of thoughts, and that if you push something in (for example sensations of the breath) something else will automatically be pushed out. Working memory does have finite attentional resources available. In line with the perceptual load theory of attention (Lavie & Tsal, 1994), a high perceptual load of attention on other material (in our study: the breath) may prevent unwanted distractors from intruding (Erskine et al., 2017). This principle may explain the observed phenomenon that focusing on the breath diminished the mental space available for distressing thoughts.

However, it may be necessary to acknowledge the troubled and anxious thoughts first. As described by participants, their uses of mindfulness included noticing and acknowledging their level of stress and troubling thought, before directing attention to the breath. According to one study, distracting oneself from distressing emotions is healthy, on the condition that the distressing emotions are first acknowledged, rather than simply just avoided (Wolgast & Lundh, 2017). Perhaps acknowledging anxious thoughts frees test-anxious students from suppressing them, thereby freeing up cognitive resources (Tempel & Neumann, 2016).

In general, disengaging from preoccupation with the threat of failure may be more difficult for test-anxious students (Rossignol et al., 2013). However, mindfulness might increase working memory capacity by reducing the tendency for thoughts to be usurped by threat. Jha et al. (2010) reported that military personnel who engaged in more mindfulness practice had increased working memory capacity under stress, suggesting that the practice reduced distraction from thoughts and feelings accompanying the stress they were under. In the present study, when shifting attention to the breath, the participants’ working memory probably became freed from containing only threatening thoughts, freeing cognitive capacity. We consider this an alternative to being swept away by, or struggling to suppress, these thoughts and feelings. Participants described this as being able to think.

### Deciding where to place attention

Mindfulness training can improve the management of attentional resources (Kang et al., 2013). When the goal is to attend to the task at hand, streams of thought that distract from the task can be seen as a type of unwanted “mind wandering” or distraction. Attention “wanders” (or flows) away from the tasks, towards some perceived danger. One might also say that attention is pulled away from the task, towards danger and discouraging thoughts such as the thought of not managing anything. The picture of a “maelstrom” describes this usurping of attention well. When attention is usurped by danger and discouraging thoughts, less attention is left for gaining academic knowledge during preparations or accessing such knowledge during exams.

Mindfulness practices train the capacity to repeatedly redirect attention towards the breath when the mind wanders towards perceived danger. Instead of letting discouraging self-referential thoughts about an imminent danger take up cognitive capacity, attentional capacity is used to attend to the breath from moment to moment. In other words: the breath functions as a benign distractor. This is in line with a study that showed that improvements in performance following mindfulness training among participants who were prone to distraction at pretesting, could be explained by a reduced occurrence of distracting thoughts during tests after the training (Mrazek et al., 2013)

### The window of tolerance

Although mindfulness instructions do not involve purposefully slowing the breath, the rate of breathing commonly slows down automatically during mindfulness meditation (Ahani et al., 2014). Slow breathing is known to reduce arousal in mice as well as humans (Yackle et al., 2017; Zaccaro et al., 2018). Research suggests that “Breath Focus Mindfulness practices” is an effective emotional regulation strategy (Zhang et al., 2019).

Some stress or arousal is useful and compatible with academic performance (Jamieson et al., 2016). A middle level of arousal where individuals can think clearly, is most compatible with performing well. Moderate levels of arousal allow an individual to “think, behave, and feel with balance and effectiveness” (Siegel, 1999, p. 253). In contrast, a level of arousal that triggers a fight or flight response is unhelpful. Many of the symptoms that participants described before the course, such as trembling, feeling faint, and nausea, can be attributed to high levels of sympathetic nerve system activity that occur when individuals are outside of their window of tolerance because of perceived danger (Siegel, 1999). Since dangers typically draw attention to them, a fixation on a danger can be difficult to break free from, like being in the headlights of a suddenly oncoming car, or in a maelstrom. One can feel unable to move and get out of harm’s way (freeze). One’s attention is spent on the frightening car or the maelstrom that draws all attention to itself.

If arousal can be lowered, the grip of danger on attention may loosen somewhat, and enable other thoughts to enter the mind. One can picture this as a “breaking up” of the flow of fearful thoughts that may allow bits of other thoughts to enter the mind and be noticed. This agrees with the descriptions of the students in this study. They said that breath-focus enabled more helpful thoughts and knowledge to catch their attention.

### Stepping out of “autopilot”

Kang et al. (2013) describe how mindfulness training may help individuals become aware of automatic and subconscious responses. Participants in this study described being able to break up habitual streams of thought by purposefully reorienting attention to the breath and then to academic tasks. This is in agreement with experimental and neurological studies that show that mindfulness training is followed by improved abilities to reorient attention, as well as better emotion regulation (e.g., Dumontheil et al., 2023).

Making active, conscious, choices of what to do and where to place attention, has been termed stepping out of autopilot in interventions such as MBSR and Mindfulness-based Relapse prevention (MBRP, Witkiewitz et al., 2014). Using mindfulness to step out of habitual ways of responding has been used for coping with diverse habits such as smoking and abusing drugs or alcohol (Vinci et al., 2023; Witkiewitz et al., 2013), unhealthy use of benzodiazepines and opiates (Dundas et al., 2020) and stress-reduction in medical students (Phang et al., 2014). The current study suggests that adolescents may use mindfulness to step out of streams of fearful thought and redirect attention to academic material.

### Buddhist notion of unwholesome thoughts

One might ask if choosing to direct attention to certain thoughts and not others, as described by the students in this study, is consistent with mindfulness. As mentioned above, mindfulness is typically described as receptive, inviting all thoughts and feelings into the mind, not pushing any thoughts or feelings away. According to Anālayo (2020), Buddhist mindfulness was at first defined as passive, nonjudging, and receptive, a *being* rather than *doing* form of attention. In line with this receptive quality, Kabat-Zinn (2021) called mindfulness a “radical non-doing” (Kabat-Zinn, 2021, p. 1034). Still, he writes that the practices may inform later choice of actions and “opens us up to the possibility of acting at least a bit more wisely in this world” (Kabat-Zinn, 2021, p. 1037).

Anālayo (2020) notes that later Buddhist discourses became more concerned with the active form of mindfulness. In that active form, attention could be actively and consciously “plunged into” “the objects in the mind” (p. 1131). “Unwholesome” thoughts and hindrances could be dismissed by changing one’s focus of attention. Students of mindfulness were encouraged to “counter the recurrent arising of associations and reflections that are of a detrimental nature” (p. 1133) by directing the mind to a wholesome topic instead. Anālayo (2020) writes:

The discourse begins with the advice that one should simply shift away from what is unwholesome and direct the mind to a wholesome topic instead. If that has not worked, one should face the unwholesome thoughts in the mind by firmly establishing a clear recognition of their detrimental nature (p. 1133).

The reduction of attention to distracting and “unwholesome” thoughts is part of both Pali and Chinese Madhyama-āgama discourses about how to train mindfulness (Anālayo, 2020). This implies that the active shifting of attention from unhelpful thoughts to more helpful thoughts is in accordance with at least some mindfulness teachings. The practitioner is encouraged to differentiate between unhelpful and helpful “objects of the mind”. Of course, what is defined as helpful and unhelpful will depend on the practitioners’ goals. For students in the current study, it was to manage exams.

### Practical implications

The study has several practical implications, and we will briefly mention some. First, participants who use breath focus to clear troubling thoughts should not be mistaken for employing thought suppression during exams. On the contrary, they may be practicing beneficial self-distracting by attending to the breath and making room for other thoughts. Second, participants should not feel that they need to be totally relaxed when preparing and sitting exams, as a middle level of arousal is more realistic for many, and in agreement with doing well on exams. Third, even in the presence of difficult thoughts and hindrances, directing attention to academic tasks during exams is feasible as long as other thoughts do not monopolize attentional resources. Lastly, when fearful thoughts threaten to overwhelm during exam preparation or sitting, redirecting attention to the breath—like in mindfulness meditation—can help break habitual patterns and prevent being carried away by fear.

### Strengths and limitations

The interviews provided rich material for exploring adolescents’ and our understanding of processes that may be involved in high-school students’ application of mindfulness for school-related stress. Perhaps the richness of the material was partly because interviews were done at a time when mindfulness was new to many Norwegian adolescents so descriptions of their experiences were in a language that had not yet absorbed all the typical mindfulness expressions and discourses. The respondents (and one of the three interviewers) were mostly unfamiliar with the expressions and conceptualizations typically used in Western mindfulness discourses, and were in a non-English language. This unfamiliarity with concepts in the field forced respondents and interviewers to specify what they meant and might have contributed to the use of words not typical of mindfulness discourses, such as pushing thoughts out of the mind and “filling the head with the breath”. We believe this was useful when looking to understand the processes that connect mindfulness to test anxiety in the current study.

Other strengths were the large number of in-depth interviews conducted by psychologists who had not taken part in the intervention and who carefully avoided advocating for mindfulness during the interviews. Moreover, participants knew that we did not know if mindfulness would be useful for their purposes and seemed highly motivated to speak about their experiences.

A limitation of this study is that there were few males and non-completers in the study, so results may not transfer to the experiences of male adolescents and non-completers. Moreover, the challenges associated with learning and applying mindfulness are not described in this article. The study does not provide evidence of how often participants used the strategies they described. Furthermore, a recent parallel randomized controlled study did not find that a mindfulness-based intervention increased adolescents’ capacity to attend to pertinent material during high affective intensity as measured through post-course experiments and self-report (Dunning et al., 2022). While feeling more confident has inherent value, other designs are needed to examine if the new strategies manifest in performance at a group level.

## Conclusion

We propose that the responses described in the current study can be understood as follows. When anxious, it is common to focus on threats (e.g., Eysenck et al., 2007). The threat may be external, or internal such as negative inner self-statements, e.g., “this will never work”. When caught up in focus on a threat, task-relevant knowledge that the adolescent might possess may be out of awareness due to limited capacity in working memory. The adolescents’ energies and attentional focus are instead directed towards handling or suppressing the threat. As the student gets caught up in an anxious train of thought, ruminates on the consequences, and ends up expecting the worst; anxiety escalates. However, participants can learn to actively relocate attention to the breath when thoughts are usurped in anxiety-provoking streams and attention drifts away from the present. Attending to sensations of the breath may function as a healthy distractor that reduces arousal and brings individuals into their window of tolerance. Some attentional resources and energy are freed from being locked into a fixation on the threat. This “freed up” attention can be used to make choices about what to attend to, providing room in the mind for ideas useful to solving the academic task. This relocation of attention likely needs to be repeated every time attention is usurped in a stream of fear during preparations and exams, just like redirecting attention to the breath is repeatedly done in other mindfulness practices.

## Supplementary Material

AppendixInterviewGuide.doc

## Data Availability

Data are not available since we have not requested permission to deposit or share them.

## Appendix Interview-guide for mindfulness when facing exams

### Introduction

In this interview, we are interested in several things: what you’ve gotten out of this course (if anything), what’s been challenging, what you’ve felt skeptical about. If there is anything you’ve learned that you wouldn’t have thought about, on your own. As well as other things you’ve experienced. We are not looking for anything in particular, so everything you can tell us, is excellent. Do you have any questions before we start?

#### Nature of school-related stresses

First, I have a few questions about stressors in high school. Not everyone knows how stressful it can be to attend high school.

1. What has been stressful for you, in relation to school?
2. How long has it been like that?
3. What have you tried earlier, to deal with this?
4. Do you do anything different now, after the course?

#### General question about their experiences with the intervention

And now I would like to ask you a general question: How was the course?

(if participants have trouble remembering, tell them that you will read the components slowly, and ask them to stop you whenever something comes to mind)

**If the participants do not cover the following areas, please ask about them. Some suggestions for probes:**

##### Probes about the usefulness in relation to the participants’ own goals:

1. Has the course been useful for you (explore. If yes: how? What has been useful about the course? If no, listen to their experiences, what might be possible reasons?)
2. Have you learned anything you wouldn’t have thought about on your own?
3. Have you experienced that the course has influenced you in relation to tests or exams? Preparations for these?
4. Are there any practices that have been more useful than others?
5. Has mindfulness influenced you in your daily life and led to any changes there?

##### Probes about self-acceptance and self-kindness

1. Do you feel that you have become more kind towards yourself. (if yes: is this good? How/how not)
2. Do you think that you’ve become more accepting of yourself?
3. Some of the practices have been to allow thoughts to come and go, without getting caught in them or evaluating them. What are your thoughts about that?
4. Have you become more aware of self-judging thoughts?

##### Probes about the “Breathing pause”

Have you used the practice of taking a breathing pause? (If yes: Has this been useful in any way. If yes: how?)

##### Probes about challenges

1. Have there been difficulties associated with mindfulness?
2. Have you been skeptical?
3. Has using mindfulness been challenging?
4. Have you had any uncomfortable experiences?
5. Was there anything you missed regarding the course?

#### Ending:

Are there other experiences you wish to add?

If you should summarize your experiences with the course with one, two, or three words, what would these words be?

(Thank the participant for completing the interview and ask: “how was this for you”?)
