# Supplementary material for: Being able to think when caught in the maelstrom - how adolescents used mindfulness when facing exams
Source: Int J Qual Stud Health Well-being. 2024 Jul 5;19(1):2375660. doi: 10.1080/17482631.2024.2375660 (PMC11229735; doi:10.1080/17482631.2024.2375660)
Supplement: AppendixInterviewGuide.doc [file ZQHW_A_2375660_SM1560.doc]

**Interview-guide for Mindfulness when facing exams**

**Introduction**

In this interview, we are interested in several things: what you’ve gotten out of this course (if anything), what’s been challenging, what you’ve felt skeptical about. If there is anything you’ve learned that you wouldn’t have thought about, on your own. As well as other things you’ve experienced. We are not looking for anything in particular, so everything you can tell us, is excellent. Do you have any questions before we start?

**I Nature of school-related stresses.**

First, I have a few questions about stressors in high school. Not everyone knows how stressful it can be to attend high school.

1) What has been stressful for you, in relation to school?

2) How long has it been like that?

3) What have you tried earlier, to deal with this?

4) Do you do anything different now, after the course?

**II General question about their experiences with the intervention.**

And now I would like to ask you a general question: How was the course?

(if participants have trouble remembering, tell them that you will read the components slowly, and ask them to stop you whenever something comes to mind)

**If the participants do not cover the following areas, please ask about them. Some suggestions for probes:**

**II.1 Probes about the usefulness in relation to the participants’ own goals:**

1. Has the course been useful for you (explore. If yes: how? What has been useful about the course? If no, listen to their experiences, what might be possible reasons?)
2. Have you learned anything you wouldn’t have thought about on your own?
3. Have you experienced that the course has influenced you in relation to tests or exams? Preparations for these?
4. Are there any practices that have been more useful than others?
5. Has mindfulness influenced you in your daily life and led to any changes there?

**II.2 Probes about self-acceptance and self-kindness**

1. Do you feel that you have become more kind towards yourself. (if yes: is this good? How/how not)
2. Do you think that you’ve become more accepting of yourself?
3. Some of the practices have been to allow thoughts to come and go, without getting caught in them or evaluating them. What are your thoughts about that?
4. Have you become more aware of self-judging thoughts?

**II.3 Probes about the “Breathing pause”**

Have you used the practice of taking a breathing pause? (If yes: Has this been useful in any way. If yes: how?)

**II.4 Probes about Challenges**

1. Have there been difficulties associated with mindfulness?
2. Have you been skeptical?
3. Has using mindfulness been challenging?
4. Have you had any uncomfortable experiences?
5. Was there anything you missed regarding the course?

**III Ending:**

Are there other experiences you wish to add?

If you should summarize your experiences with the course with one, two, or three words, what would these words be?

(Thank the participant for completing the interview and ask: “how was this for you”?)
